# ISFM-SLAM: dynamic visual SLAM with instance segmentation and feature matching

**DOI:** 10.3389/fnbot.2024.1473937

**Published:** 2024-11-20

**Authors:** Chao Li, Yang Hu, Jianqiang Liu, Jianhai Jin, Jun Sun

**Affiliations:** ^1^School of Artificial Intelligence and Computer Science, Jiangnan University, Wuxi, China; ^2^China Ship Scientific Research Center, Wuxi, China

**Keywords:** simultaneous localization and mapping (SLAM), instance segmentation network, dynamic environment, motion consistency detection, feature matching

## Abstract

**Introduction:**

Simultaneous Localization and Mapping (SLAM) is a technology used in intelligent systems such as robots and autonomous vehicles. Visual SLAM has become a more popular type of SLAM due to its acceptable cost and good scalability when applied in robot positioning, navigation and other functions. However, most of the visual SLAM algorithms assume a static environment, so when they are implemented in highly dynamic scenes, problems such as tracking failure and overlapped mapping are prone to occur.

**Methods:**

To deal with this issue, we propose ISFM-SLAM, a dynamic visual SLAM built upon the classic ORB-SLAM2, incorporating an improved instance segmentation network and enhanced feature matching. Based on YOLACT, the improved instance segmentation network applies the multi-scale residual network Res2Net as its backbone, and utilizes CIoU_Loss in the bounding box loss function, to enhance the detection accuracy of the segmentation network. To improve the matching rate and calculation efficiency of the internal feature points, we fuse ORB key points with an efficient image descriptor to replace traditional ORB feature matching of ORB-SLAM2. Moreover, the motion consistency detection algorithm based on external variance values is proposed and integrated into ISFM-SLAM, to assist the proposed SLAM systems in culling dynamic feature points more effectively.

**Results and discussion:**

Simulation results on the TUM dataset show that the overall pose estimation accuracy of the ISFM-SLAM is 97% better than the ORB-SLAM2, and is superior to other mainstream and state-of-the-art dynamic SLAM systems. Further real-world experiments validate the feasibility of the proposed SLAM system in practical applications.

## 1 Introduction

Simultaneous Localization and Mapping (SLAM) is a technology that enables robots to determine their location and construct a map in real time by collecting data in an unknown environment. The mainstream categories of the SLAM technologies include laser SLAM and visual SLAM. Due to its higher precision than the laser SLAM and acceptable construction cost, visual SLAM has become a research focus in the field of SLAM (Taketomi et al., 2017).

Feature point method and direct method are two common methods used in visual SLAM to extract information from the scene and estimate camera motion (Taketomi et al., 2017). The feature-based method represents features in the scene by extracting key points and descriptors from pixels. Davison et al. (2007) proposed a monocular visual SLAM algorithm, namely MonoSLAM, to achieves online localization and mapping by extracting and tracking feature points. ORB-SLAM2 (Mur-Artal and Tardós, 2017) utilizes feature points and descriptors with selection and scale invariance to generate camera poses, and employs closed-loop detection to optimize map consistency, thereby eliminating cumulative camera errors. On the contrary, the direct method does not rely on feature extraction or descriptor matching, but utilizes original pixel values for information extraction and motion estimation. An example is the LSD-SLAM (Engel et al., 2014), which uses image continuity information and dense optical flow fields to estimate the motion of the camera. However, the SLAM algorithms based on the aforementioned two kind of methods usually treats the external environment as static, ignoring the impact of dynamic objects on map accuracy. In a dynamic environment, the movement of objects may cause changes in the map, making it difficult for traditional visual SLAMs to accurately estimate the camera motion and scene structure. Hence, new visual SLAM algorithms need to be developed to handle the issues in dynamic environments.

In recent years, scholars have focused on the combination of deep neural networks (DNNs) and visual SLAM to achieve good SLAM effects in dynamic environments. DNNs can provide semantic information for SLAM systems, enhancing the systems’ perception capabilities and effectively improves the accuracy of both tracking and mapping. For instance, Yu et al. (2018) proposed the DS-SLAM, which integrates the semantic segmentation network SegNet (Badrinarayanan et al., 2015) and motion consistency detection into the ORB-SLAM2. By eliminating feature points associated with dynamic objects, this approach mitigates the adverse effects of dynamic environments and improves the stability of map construction. The DynaSLAM (Bescos et al., 2018) employs mask Region-based Convolutional Neural Network (R-CNN) and multi-view geometry to filter dynamic feature points by integrating sparse and dense map information. However, both DS-SLAM and DynaSLAM are prone to issues such as insufficient or incorrectly removed feature points, often due to erroneous prior knowledge or challenging lighting conditions, which can ultimately compromise SLAM accuracy. Detect-SLAM (Zhong et al., 2018) incorporated a DNN-based object detector into the ORB-SLAM2 system and added three new modules: moving object rejection, object mapping, and SLAM-enhanced detector. This algorithm enhances the accuracy of localization and mapping in a highly dynamic environment, but its performance is not as robust as that of ORB-SLAM2 in a static environment. As demonstrated, the aforementioned visual SLAM systems mitigate interference from dynamic objects by discarding feature points associated with them. However, in certain scenarios, such approaches may mistakenly remove feature points from static objects or inadvertently retain feature points from dynamic objects. This often results in a reduction in the number of feature points matches, consequently causing the SLAM systems to lose track. Thus, addressing the issue of incorrect feature point matching in dynamic environments remains a pressing challenge for visual SLAM systems.

Several research have been conducted in response to the above problem. For example, Cai and Wu (2022) proposed a SLAM algorithm based on the YOLACT (Bolya et al., 2019) instance segmentation network. This SLAM performed static point recovery based on external constraints after removing dynamic objects, which to some extent alleviated the problem of insufficient feature points. However, YOLACT’s bounding box loss primarily emphasizes the coordinates of the four corners rather than the position of the center point, which can lead to a shift in the segmented bounding box. Moreover, the bounding box loss does not fully take into account the shape and size of the target object, potentially resulting in suboptimal segmentation accuracy. Integrated with the SegNet, Cui and Ma (2019) proposed the SOF-SLAM to tightly couple visual semantic information and optical flow information, thereby effectively and reasonably removing dynamic feature points. Based on the ORB-SLAM2, Su et al. (2022) developed a parallel semantic module based on the lightweight object detection network YOLOv5s. This module utilizes semantic information to optimize the homography matrix, and uses optical flow masks to remove dynamic feature points from the image. He et al. (2023) proposed OVD-SLAM, which integrates semantic, depth, and optical flow information to differentiate between foreground and background, thereby identifying dynamic objects. It can be observed that combining semantic segmentation with optical flow for detecting dynamic feature points has become a prominent research focus. However, optical flow estimation algorithms are prone to errors when handling fast motion and occlusions. Fast-moving objects can cause instability in optical flow estimation, while occluded objects may lead to distorted optical flow, both of which can negatively impact segmentation accuracy.

To overcome the challenges of suboptimal instance segmentation accuracy and incorrect feature point classification in dynamic visual SLAM systems, this paper proposes ISFM-SLAM, a dynamic visual SLAM system based on ORB-SLAM2, which incorporates an improved instance segmentation network, a novel motion consistency detection approach, and an introduced efficient learned binary image descriptor. The main contributions of this work are as follows:

1. Accurately obtaining prior knowledge of objects in an image is crucial for designing an effective visual SLAM system. To this end, we propose an improved instance segmentation network based on YOLACT and integrate it into the ISFM-SLAM system. Specifically, we replace YOLACT’s backbone with Res2Net-50 (Gao et al., 2019), which offers a superior receptive field, a more compact scale, and greater ease of deployment. Furthermore, we introduce CIoU_Loss (Zheng et al., 2020) to rectify YOLACT’s occasional inaccuracies in bounding box estimation. In this way, the accuracy of the instance segmentation network is significantly enhanced, which greatly benefits subsequent processes such as feature point matching.
2. To address the issue of incorrectly removing or retaining feature points in certain scenarios, this paper introduces a novel motion consistency detection approach based on the Perspective-n-Point (PnP) algorithm (Lepetit et al., 2009). By calculating the difference in external parameters between frames, the PnP-based motion consistency detection method can more reliably determine whether feature points belong to the same static object. This enables more accurate removal of dynamic and incorrectly matched feature points, while ensuring the proper retention of static feature points, ultimately leading to a more reliable motion consistency detection outcome.
3. Moreover, a learned binary image descriptor, BEBLID (Suárez et al., 2020), is combined with ORB key point detection to further enhance the accuracy and efficiency of feature matching. The BEBLID descriptor, trained using a boosted method, significantly improves feature point matching accuracy, and its parallel computing capability ensures high computational efficiency. This allows feature matching based on the BEBLID descriptor to maintain high accuracy even in scenes with numerous dynamic objects, while also better satisfying the real-time performance requirements of SLAM systems in practical applications.
4. Simulation results demonstrate that the proposed ISFM-SLAM system achieves outstanding overall pose estimation accuracy in both low-dynamic and high-dynamic environments, with a 97% improvement compared to the baseline ORB-SLAM2, and outperforms many other mainstream and state-of-the-art dynamic SLAM algorithms. Furthermore, real-world experimental results validate the high accuracy of ISFM-SLAM in both dynamic feature point removal and feature point matching.

The remainder of this paper is organized as follows. Section 2 reviews the related work pertinent to the studies presented in this paper. Section 3 presents the proposed ISFM-SLAM system, including the details of the improved instance segmentation network, PnP-based motion consistency detection, and BEBLID feature matching. Simulation and real-world experimental results, along with corresponding discussions, are presented in Sections 4 and 5, respectively. Some conclusions and directions for future work are provided in Section 6.

## 2 Related work

### 2.1 ORB-SLAM2

As a classic visual SLAM system, ORB-SLAM2 is composed of three main parallel threads: tracking, local mapping, and loop closure (Mur-Artal and Tardós, 2017). To locate the camera pose and generate keyframes, the tracking thread extracts feature points from each frame of images and matches them with the local map. The local mapping thread receives the keyframes from the tracking thread, uses the bundle adjustment (BA) algorithm to optimize the camera pose, and eliminates redundant information from the map. The loop closure thread detects the map loop, corrects the accumulated drift, and eliminates accumulated errors. After optimizing the pose graph, the ORB-SLAM2 launches the fourth thread to perform full BA, to calculate the optimal structure and the motion solution. For a detailed explanation of the ORB-SLAM2 system framework and its components, refer to Mur-Artal and Tardós (2017).

### 2.2 YOLACT instance segmentation network

Instance segmentation is a task in the field of computer vision that aims to identify the pixel-level segmentation of each object in an image and assign a unique identifier to each object. Instance segmentation generates a mask on the image target, but preserve the shape and features of the target. The YOLACT network is a one-stage instance segmentation model proposed by Bolya et al. (2019). Compared with the two-stage models represented by Mask R-CNN (He et al., 2017), the YOLACT has the advantages of fewer parameters and faster operation, making it more suitable for application in SLAM systems with high real-time requirements.

In the YOLACT instance segmentation network, a backbone network based on ResNet-101 is used to extract multi-scale feature maps from the input image. These feature maps are then passed through a feature pyramid network for further processing, leading to the prediction of bounding boxes. To evaluate the regression performance of the model for the location of the bounding box, YOLACT uses Smooth L1 as the bounding box regression loss function. After regression, the detected boxes are filtered by non-maximum suppression (Qiu et al., 2018) to obtain the instances corresponding to each object, and the mask segmentation results corresponding to each anchor are generated by linear combination. The specific steps of YOLACT instance segmentation are detailed in Bolya et al. (2019).

## 3 Methodology

### 3.1 ISFM-SLAM framework based on ORB-SLAM2

**Figure 1:**
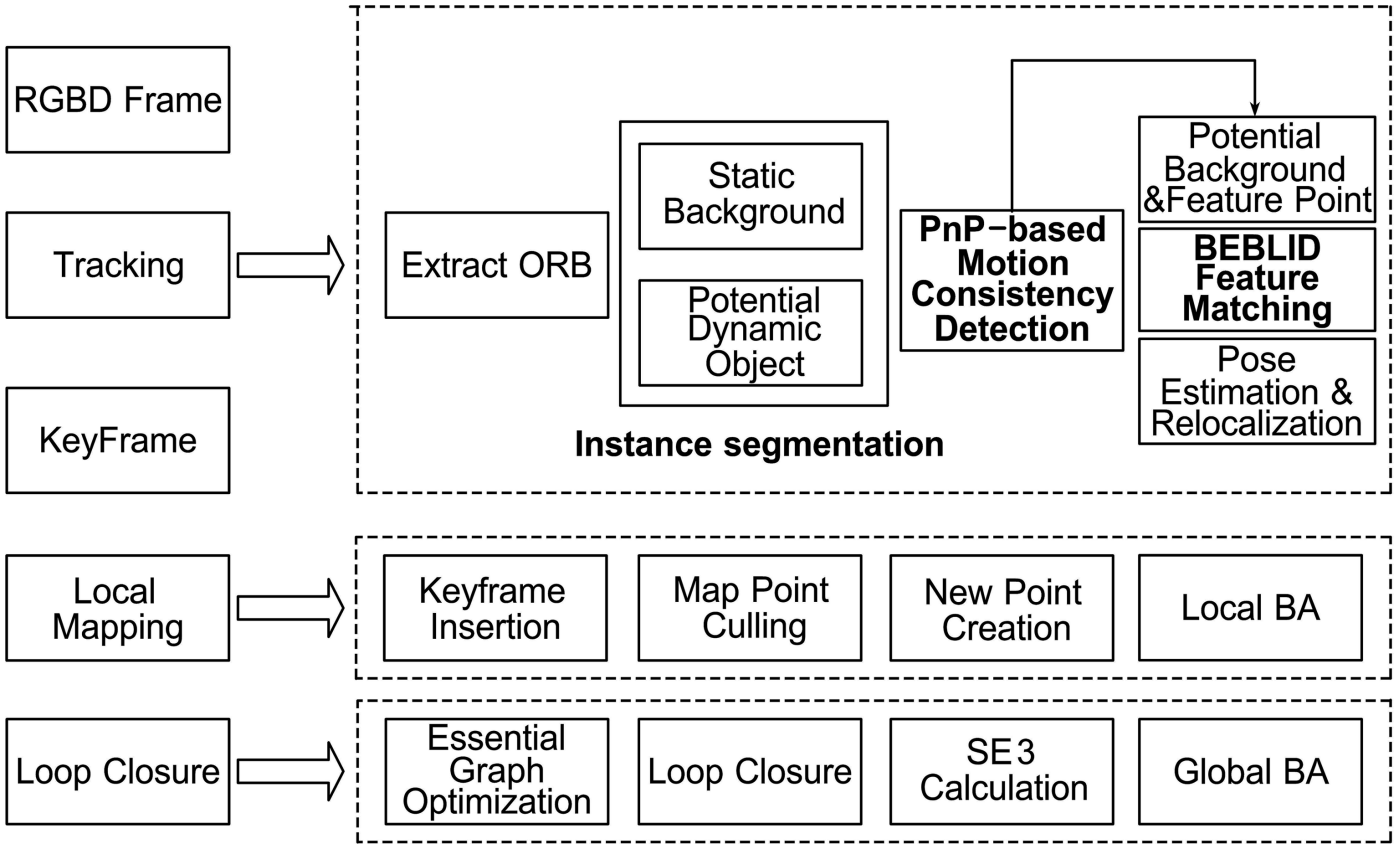
Overall pipeline of the ISFM-SLAM system.

The key to enhancing ORB-SLAM2 in dynamic environments is to accurately perform instance segmentation on the image, enabling the reasonable removal of feature points associated with dynamic objects. To this end, we modified the tracking thread in the ORB-SLAM2 system in this paper to construct the ISFM-SLAM framework. Specifically, an improved instance segmentation network is introduced in the tracking thread to divide the image frame into static background and potential dynamic instances. Then, a motion consistency detection method based on PnP is employed to effectively remove dynamic instances while retaining static feature points. Finally, a feature matching algorithm based on the boosted efficient BEBLID descriptor is utilized to perform feature matching and accurately calculate the camera pose. Following the completion of the tracking thread, ISFM-SLAM proceeds with local mapping, loop closure, and global BA, ultimately producing the corresponding poses and a global point cloud map. The overall pipeline of the ISFM-SLAM system is illustrated in Figure 1.

### 3.2 Improved instance segmentation network based on Res2Net and CIoU_Loss

In order to improve the accuracy of the YOLACT segmentation network, this paper utilizes the Res2Net-50 to replace the backbone of the original YOLACT, so that the multi-scale receptive field of the network can be improved. Res2Net, proposed by Gao et al. (2019), is a multi-scale backbone network for computer vision tasks such as object detection and semantic segmentation. By constructing layered residual connections in the convolutional blocks, Res2Net-50 is capable of representing multi-scale features within a single residual block. This allows the network to better capture image features at different scales, thus improving the accuracy of instance segmentation. Moreover, Res2Net-50 can enhance the network’s ability to comprehensively learn image information by expanding the receptive field of each layer, resulting in more precise localization and segmentation of target objects. Another advantage of Res2Net-50 is its ease of integration into existing state-of-the-art CNN models, offering flexibility that allows it to excel across various tasks and improve the overall performance of the YOLACT model. Given the high real-time requirements of the SLAM algorithm in dynamic environments, this paper implements Res2Net-50 with a scale of 4 as the backbone network of the improved instance segmentation network, which can not only ensure sufficient semantic features but also reduce the cost of instance segmentation. The detailed architecture of the adopted Res2Net-50 backbone can be found in Gao et al. (2019).

In addition, the CIoU_Loss function (Zheng et al., 2020) is used to replace the original loss function Smooth L1 in the original YOLACT to obtain higher accuracy of bounding box regression. The reason is that the Smooth L1 function cannot accurately measure the position of the predicted box due to the lack of calculation of the intersection over union (IoU) and the minimum bounding rectangle. In contrast, CIoU_Loss considers not only the overlap area between the predicted box and the ground truth box, but also the distance between their center points and the aspect ratio, which are geometric factors critical for accurately localizing and segmenting the target object. By introducing these geometric elements, CIoU_Loss can more effectively handle challenging localization scenarios, resulting in better regression performance for the predicted box compared to Smooth L1. Another reason we use CIoU_Loss is that this loss function is its faster convergence during training, which improves the model’s training efficiency. The calculation method for CIoU_Loss is shown in Equation (1):

(1) $$\left\{ \begin{array}{c} \begin{array}{c} L_{\mathrm{CIoU}}=1-IoU\left(b,b^{gt}\right)+\frac{2\rho \left(b,b^{gt}\right)}{c^{2}}+\alpha \nu ,\\ IoU\left(b,b^{gt}\right)=\frac{\left|b⊥b^{gt}\right|}{\left|b\cup b^{gt}\right|}, \end{array}\\ \begin{array}{c} \alpha =\frac{\nu }{\left(1-P_{IoU}\right)+\nu },\\ \nu =\frac{4}{\pi^{2}}{\left(\arctan\frac{w^{gt}}{h^{gt}}-\arctan\frac{w}{h}\right)}^{2}\text{,} \end{array} \end{array}\right.$$

where ρ represents the distance between the geometric centers of the prediction box b and the target box $b^{gt}$ . $\frac{w^{gt}}{h^{gt}}$ and $\frac{w}{h}$ represent the aspect ratios of the target box and prediction box, respectively. c represents the diagonal length of the smallest circumscribed rectangle of the prediction box and target box. With the implementation of the CIoU_Loss, the convergence speed and the multi-scale object detection robustness of the improved instance segmentation network can be significantly enhanced.

### 3.3 PnP-based motion consistency detection

The instance segmentation network is capable of acquiring prior information for the motion of objects within a video frame. However, relying solely on this prior information to determine whether a feature point should be removed may lead to two issues. One is the feature points of objects in a stationary state with non-dynamic prior information, such as those of a stationary person, will be removed. Another one is the feature points of moving objects with non-dynamic prior information will be preserved, such as those of the books that interact with people. Thus, it is necessary to compare the motion state of the same object across consecutive frames to accurately decide whether the associated feature points should be removed. That is, a motion consistency detection method should be employed to assist in the classification of feature points.

To improve the performance of consistency detection of motion objects, this paper proposes a novel motion consistency detection method based on the PnP algorithm. On the premise that the camera has observed the 3-dimensions (3D) positions of multiple points, the PnP algorithm accurately determines the position and orientation of feature points in 3D space by solving the geometric relationships between the camera and the feature points in the scene. The detailed procedure of the PnP algorithm can be found in Lepetit et al. (2009). Our method analyzes this geometric relationship to compare changes in feature points across frames, thereby determining whether the feature points belong to the same static object. If the motion trajectories of the feature points are inconsistent, they may be either incorrectly matched or dynamic feature points, and will thus be appropriately removed. In this way, the PnP algorithm is employed to detect the motion consistency of feature points across consecutive frames, enabling a more accurate distinction between true dynamic and static feature points, even in the presence of inaccurate prior information. The specific steps of the PnP-based motion consistency detection method are as follows.

After applying the improved instance segmentation network proposed in Section 3.2 to divide video frames into static background and potential dynamic instances, we use PnP algorithm to calculate the static baseline extrinsic parameter $T_{\mathrm{static}}$ based on the continuous two frame $F_{n-1}$ and $F_{n}$ under constant speed motion model as shown in Equation (2):

(2) $$T_{\mathrm{static}}=PnP\left(P_{\mathrm{static},n},\;P_{\mathrm{static},n-1}\right)$$

where $P_{\mathrm{static},n}$ and $P_{\mathrm{static},n-1}$ denotes the sets of the static background feature points in $F_{n}$ and $F_{n-1}$ , respectively.

Using $T_{\mathrm{static}}$ as the baseline, it is capable to determine the true dynamic instances among the potential dynamic instances in the current frame. Specifically, for each potential dynamic instance, its corresponding pose transformation matrix $T_{i}$ can be calculated using Equation (3):

(3) $$T_{i}=PnP\left(P_{i,n},P_{i,n-1}\right)$$

where $P_{i,n}$ and $P_{i,n-1}$ are the sets of the feature points of instance i in $F_{n}$ and $F_{n-1}$ , respectively. Then, the 2-norm of the difference matrix $A_{i}$ between $T_{\mathrm{static}}$ and $T_{i}$ can be calculated by Equation (4):

(4) $$A_{i}=∥T_{\mathrm{static}}-T_{i}{∥}_{2}$$

If $A_{i}$ is greater than the perset threshold $T_{d}$ , then the potential instance i is determined to be a true dynamic instance.

Based on the above procedure, the ORB feature points belonging to the dynamic instance can be discarded more accurately. After that, the remaining static ORB feature points can be utilized to calculate the camera pose, and then the stable and accurate extrinsic parameters can be obtained.

### 3.4 BEBLID feature matching

Since feature matching plays a pivotal role in visual SLAM, the accuracy and efficiency of feature matching algorithm directly influences the quality of subsequent localization and mapping. ORB-SLAM2 employs binary robust independent elementary features (BRIEF) to obtain descriptors of feature points. However, the expressiveness of BRIEF descriptors is constrained by their derivation from straightforward pixel comparison, diminishing the matching accuracy of the ORB algorithm. In addition, although the improved instance segmentation network and PnP-based motion consistency detection can largely prevent the incorrect removal of feature points, in cases where a frame contains a significant number of dynamic instances, the available points for feature matching may become insufficient due to the elimination of dynamic feature points, thereby affecting the overall performance of the SLAM system. To address this, the ISFM-SLAM framework implements a feature matching algorithm based on the BEBLID descriptor (Suárez et al., 2020). BEBLID employs a learning-based approach, specifically adaptive boosting (AdaBoost) (Pardoe and Stone, 2010), for training. AdaBoost combines multiple weak classifiers, iteratively adjusting the weights of samples to focus more on previously misclassified instances, thereby significantly improving classification accuracy. The use of the AdaBoost algorithm to minimize the BEBLID loss function is described in Equation (5):

(5) $$L_{\mathrm{BEBLID}}=\overset{N}{\underset{i=1}{\sum }}\exp\left(-\gamma l_{i}\overset{K}{\underset{k=1}{\sum }}\alpha_{k}h_{k}\left(x_{i}\right)h_{k}\left(y_{i}\right)\right)$$

where γ is the learning rate. $\left\{x_{i},y_{i}\right\}$ is a training set composed of pairs of image patches. $l_{i}$ is the label of the training sample. $l_{i}=1$ denotes that both patches correspond to the same image structure, while $l_{i}=-1$ denotes that they correspond to different image structures. $h_{k}\left(z\right)\text{≡}h_{k}\left(z;,f;,T\right)$ denotes the k th weak learner with weight $\alpha_{k}$ , which depends on a feature extraction function $f\left(\cdot \right)$ and a threshold T as shown in Equation (6):

(6) $$h\left(x;\;f;\;T\right)=\{\begin{array}{c} +1,\;\mathrm{if}\;f\left(x\right)⩽T\\ -1,\;\mathrm{if}\;f\left(x\right)>T \end{array}$$

In particular, the key to improving the efficiency calculation of the BEBLID descriptor is the choice of $f\left(x\right)$ . Here, $f\left(x\right)$ is defined as the average gray difference between pixels in two different image boxes as shown in Equation (7).

(7) $$f\left(x;\;p_{1};\;p_{2};\;s\right)=\frac{1}{s^{2}}\underset{q\in R\left(p_{1},s\right)}{\sum }I\left(q\right)-\underset{r\in R\left(p_{2},s\right)}{\sum }I\left(r\right)$$

where $I\left(t\right)$ is the gray value at pixel t and $R\left(p,s\right)$ is the square box with a side length of s centered at pixel p . The descriptor of the response map is shown in Equation 8.

(8) $$D\left(x\right)=A^{\frac{1}{2}}h\left(x\right)={\left[\sqrt{\alpha_{1}}gh_{1}\left(x\right)L\sqrt{\alpha_{k}}gh_{k}\left(x\right)\right]}^{T}$$

where $A=\mathrm{diag}\left(\alpha_{1},\alpha_{2},\cdots ,\alpha_{k}\right)$ .

**Figure 2:**
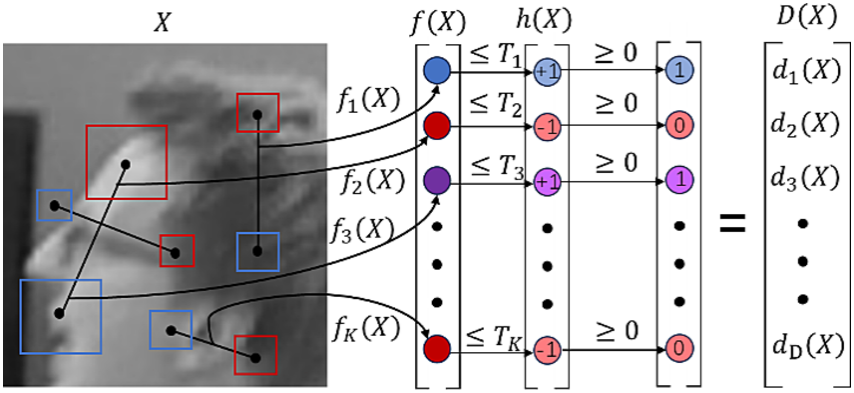
BEBLID descriptor extraction workflow (Suárez et al., 2020).

It can be seen that the BEBLID descriptor improves the loss function by using all the weak learner and the integral image, enabling the feature matching algorithm to obtain high-quality binary descriptors. As a result, feature matching based on BEBLID is more accurate than that based on BRIEF, allowing the algorithm to perform precise matching even when the number of available feature points is limited. Furthermore, BEBLID ensures relatively high feature matching accuracy under challenging lighting conditions, such as strong or weak light, thereby further enhancing the proposed SLAM system’s ability to handle complex scenes. In addition, BEBLID computes each descriptor in parallel, which can significantly improve the efficiency of feature matching. The extraction workflow of the BEBLID descriptor is demonstrated in Figure 2.

## 4 Simulation experiments and discussions

### 4.1 Simulation environment

To verify the effectiveness of ISFM-SLAM and the proposed components, a series of simulation experiments were conducted. To accelerate the training of the deep learning model, the proposed improved instance segmentation network was trained on a server with an Intel Xeon Silver 4214R CPU, 90 Giga-Bytes (GB) memory, and an RTX 3080 TI GPU (12 GB graphics memory). All the other experiments were conducted on a personal computer (PC) with the following configurations: an AMD Ryzen 75800H 3.2 GHz CPU, 16 GB memory, an RTX 3060 laptop GPU with 6 GB graphics memory. The operating system is Ubuntu 18.04 with CUDA 11.3 and Pytorch 1.11.0. The code for the improved instance segmentation network of the ISFM-SLAM is written in Python 3.6, while the codes for the other parts of the ISFM-SLAM are written in C++.

### 4.2 Performance of improved instance segmentation network

The effectiveness of visual SLAMs depends heavily on the performance of instance segmentation networks. Therefore, in this section, we analyzed the segmentation performance on some samples of the original YOLACT and the improved instance segmentation network proposed in this paper, and also compared the statistical results on public datasets by these two networks as well as some canonical and state-of-the-art instance segmentation methods. The improved instance segmentation network was trained on the COCO Minitrain dataset (Samet et al., 2020). This dataset is a subset of the Microsoft Common Objects in COntext (MS COCO) dataset (Lin et al., 2014), which contains approximately 25,000 images and all the 80 categories of the MS COCO. The Res2Net pre-trained weights were used for training the improved YOLACT network. The batch size was set to 24. The number of iterations was 100,000. Stochastic gradient descent (SGD) was utilized as the optimizer, with an initial momentum of 0.9, a learning rate of 0.001, and a weight decay coefficient of 0.0001.

**Figure 3:**
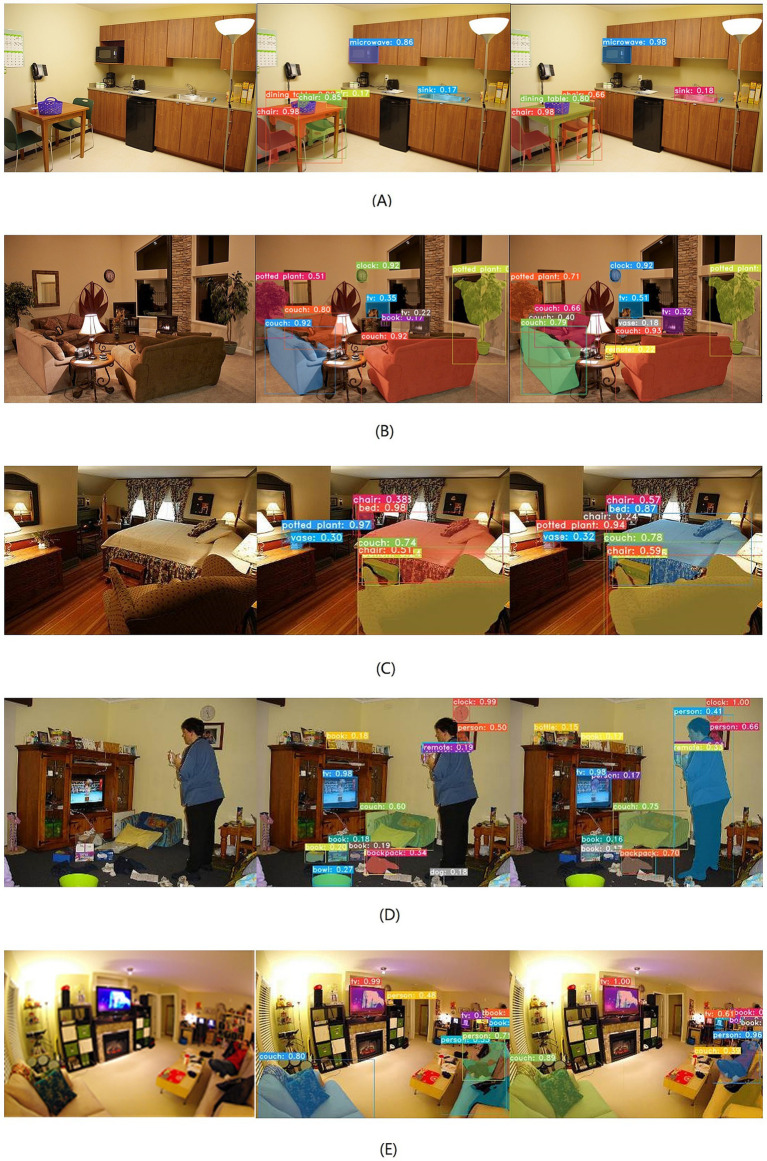
Comparison of segmentation results between our improved algorithm and YOLACT. The raw images were obtained from the COCO Minitrain Dataset, and this dataset is licensed under a Creative Commons Attribution 4.0 License (https://cocodataset.org/#termsofuse).

To facilitate a more intuitive comparison of the instance segmentation effect of the original YOLACT and the improved one, we visualize the segmentation result obtained by the two compared network on five samples with complex indoor environments of COCO dataset. The results are presented in Figure 3, where the left column contains the input images, the middle column contains the segmentation results obtained by the original YOLACT, and the right column contains the segmentation result obtained by the improved instance segmentation network. According to the results in the middle column, the original YOLACT may yield unsatisfactory outcomes in highly complex scenes. For example, an instance may be divided into two parts, as seen with the chair in Figure 3A. Additionally, some instances may not be detected or segmented, such as the vase in Figure 3B, the chair in Figure 3C, and the person in Figure 3D. Misidentification of instances can also occur, as observed with the debris on the ground and bowls on the cabinet in Figure 3D, and the sofa in Figure 3E. Furthermore, some masks may fail to accurately cover the corresponding instances, such as the person in Figure 3E. The main reasons for these issues are the poor feature extraction ability of the instance segmentation network, which leads to a lack of multi-scale perception of the scene and low localization accuracy of the detection boxes. For our proposed network, the multi-scale receptive field has been increased due to the replacement of the backbone, and the precision of the detection box has been improved because of the optimization of the boundary regression box. Consequently, the improved network can better utilize environmental semantic information and achieve more accurate segmentation, as shown by the results on the right column in Figure 3.

To demonstrate the performance of the improved network in various instance segmentation tasks, we compared our proposed network with YOLACT and some other mainstream segmentation networks, including Mask R-CNN (He et al., 2017), PolarMask (Xie et al., 2020), and FourierNet (Riaz et al., 2021), on the COCO validation set. The results are recorded in Table 1. The evaluation metrics based on the Average Precision (AP) was utilized for evaluation, including mean AP (mAP), AP50, AP75, APS, APM, and APL, where AP represents the area under the precision-recall curve for a given class. The equations for calculating AP and mAP are shown in Equations (9) and (10):

(9) $$AP=\overset{1}{\underset{0}{\int }}P\left(r\right)dr$$

(10) $$\mathrm{mAP}=\frac{\Sigma AP}{N}$$

where P is the average precision value for the current class, and N is the number of sample categories in the dataset. AP50 and AP75 are special cases of calculating AP where the IoU thresholds are set to 0.5 and 0.75, respectively. A prediction is considered correct when the IoU is greater than or equal to a certain threshold (e.g., 0.5 or 0.75). The last three metrics measure the performance of detecting objects of different scales: small, medium, and large. In addition, the frames per second (FPS) is also measured to show the efficiency of the compared segmentation methods.

**Table 1:** Performance comparison of different instance segmentation methods on COCO validation set.

Model	FPS	mAP	AP50	AP75	APS	APM	APL
Mask R-CNN	8.6	37.52	58.86	40.26	16.71	39.82	54.34
PolarMask	17.2	30.63	50.81	31.89	12.74	33.73	45.29
FourierNet	26.6	32.97	55.47	33.82	15.52	35.15	46.38
YOLACT	45	29.82	48.53	31.23	9.98	31.35	47.76
Ours	42.2	33.61	56.24	36.26	16.47	36.21	49.82

Compared with the original YOLACT, our enhanced model results in a 2.8 frames reduction in FPS, but a 3.8% improvement in mAP. This verifies that our improved model significantly enhances segmentation precision with only a slight reduction in computational speed compared to the original YOLACT. When our proposed method is compared with the other approach in Table 1, it can be seen that the efficiency of our method is much better than other competitors, and all the AP-related results are the second-best ones, only slightly worse than those by the Mask R-CNN. It should be noted that since the real-time processing capability is crucial for SLAM problems, the Mask R-CNN network may be not suitable for these applications. Therefore, it can be concluded that the proposed improved instance segmentation network can better meet the accuracy and real-time requirements of a visual SLAM system in a dynamic environment when compared to most of the other instance segmentation methods.

### 4.3 Performance analysis of the ISFM-SLAM

In this section, the absolute trajectory error (ATE) is utilized to evaluate the performance of the proposed SLAM system and the compared ones for each run. The ATE is calculated by subtracting the ground-truth from the estimated value of the camera pose, as shown in Equation 11, so that this metric can provide an intuitive representation of the accuracy of the trajectory.

(11) $$e_{\mathrm{ATE}}=\frac{1}{\;N}\sqrt{\overset{N}{\underset{i=1}{\sum }}∥T_{i}^{w}-{{\hat{T}}_{i}^{w}}_{2}^{2}∥}$$

where N is the total number of frames, ${\hat{T}}_{i}^{w}$ denotes the estimated pose trajectory, $T_{i}^{w}$ is the gound-truth trajectory, and $e_{\mathrm{ATE}}$ is the absolute trajectory error. After multiple SLAM experiments, the mean, standard deviation (STD), and Root Mean Squared Error (RMSE) of the $e_{\mathrm{ATE}}$ is adopted to evaluate the performance of SLAM systems from a statistical perspective, where the RMSE is more sensitive to occasional errors than the other metrics and thus can better reflect the robustness of the system.

Firstly, the proposed ISFM-SLAM is quantitatively compared with its baseline, ORB-SLAM2 (Mur-Artal and Tardós, 2017), using four high-dynamic sequences (labeled “walking”) and four sets of low-dynamic sequences (labeled “sitting”) from the TUM dataset (Sturm et al., 2012). Each experiment was performed three times, and the mean, STD and RMSE results obtained by the two compared SLAM systems are recorded in Table 2.

**Table 2:** Comparison of the mean, STD and RMSE of ATE obtained by the ORB-SLAM2, OVD-SLAM, and the ISFM-SLAM.

Scene	ORB-SLAM2			ISFM-SLAM			Improving rate (%)		
	RMSE	STD	Means	RMSE	STD	Means	RMSE	STD	Means
fr3/walking_static	0.3775	0.1657	0.3392	0.0081	0.0034	0.0072	97.9	97.9	97.9
fr3/walkig_xyz	0.6783	0.3761	0.5645	0.0164	0.0089	0.0137	97.6	97.6	97.6
fr3/walking_rpy	0.7565	0.3360	0.6778	0.0301	0.0161	0.0254	96.0	95.2	96.3
fr3/walking_half	0.4699	0.2458	0.4004	0.0246	0.0131	0.0208	94.8	94.7	94.8
fr3/sitting_static	0.0094	0.0045	0.0082	0.0064	0.0035	0.0061	25.5	22.2	25.6
fr3/sitting _xyz	0.0089	0.0042	0.0078	0.0103	0.0047	0.0091	−15.7	−11.9	−16.7
fr3/sitting _rpy	0.0197	0.0109	0.0163	0.0162	0.0090	0.0556	17.8	17.4	16.6
fr3/sitting _half	0.0385	0.0194	0.0338	0.0175	0.0089	0.0151	54.5	54.1	55.3

As illustrated in Table 2, the accuracy and robustness of the proposed SLAM system is significantly better than the ORB-SLAM2 in high-dynamic scenes, with an average improvement of 96.7% in mean ATE, 96.9% in STD, and 97.2% in RMSE. However, the proposed algorithm cannot obtain significantly better performance than the ORB-SLAM2 in low-dynamic scenes. Specifically, in the fr3/sitting_xyz scene, inaccurate matching or segmentation occurred in our system, which results in a decrease in accuracy. For fr3/sitting_static and fr3/sitting_rpy, since ORB-SLAM2 has already applied RANSAC to successfully remove some outliers, the advantage of the ISFM-SLAM is not very obvious. Nevertheless, the performance of the ISFM-SLAM is still outstanding in some low-dynamic scenes. For example, in the fr3/sitting_half scene including some moving instances, our proposed algorithm improves by more than 50% compared to ORB-SLAM2.

**Figure 4:**
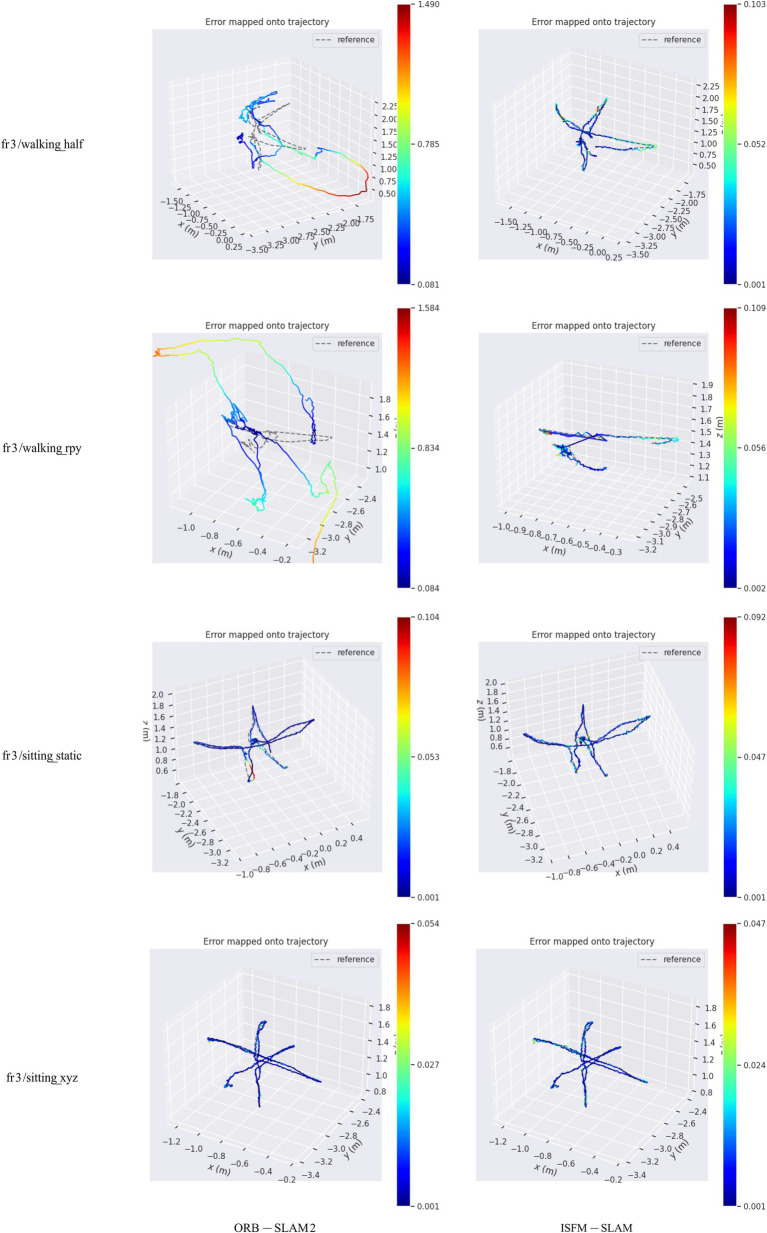
Comparison of the estimated trajectories by ORB-SLAM2 and ISFM-SLAM with the real trajectory on different sequences.

To further demonstrate the advantage of the ISFM-SLAM over ORB-SLAM2, the camera estimation trajectories obtained by the two competitors were compared with the real trajectories in four scenes including fr3/walking_half, fr3/walking_rpy, fr3/sitting_static, and fr3/sitting_xyz. The results are presented in Figure 4. From this figure, it is evident that in the high-dynamic environments, the pose trajectory estimated by the ISFM-SLAM is much more closely aligned with the real trajectory than that by the ORB-SLAM2, while in the low-dynamic environments, the two estimated trajectories are both close to the real one.

Finally, the proposed ISFM-SLAM is compared with some other dynamic SLAM systems, including Dyna-SLAM (Bescos et al., 2018), DS-SLAM (Yu et al., 2018), MR-SLAM (Sun et al., 2017), DRSO-SLAM (Yu et al., 2021), and OVD-SLAM (He et al., 2023) to verify its effectiveness. Among them, Dyna-SLAM, DS-SLAM, and OVD-SLAM is designed based on semantic segmentation approaches, MR-SLAM is implemented based on optical flow method, and DRSO-SLAM is based on both the semantic segmentation and optical flow schemes. The RMSE results of the ATE obtained by these compared SLAM systems are illustrated in Table 3, with the best results on each scene highlighted in bold. Note that except for the experimental results of the ISFM-SLAM, the results of the other compared algorithms are all from the corresponding references, and the “None” in Table 3 indicates that the corresponding sample was not tested. As shown in Table 3, the proposed is comparable to Dyna-SLAM (Bescos et al., 2018) in terms of pose estimation accuracy in high-dynamic scenes, but is superior to the other competitors. In low-dynamic scenes, the ISFM-SLAM still has a significant advantage over the other four algorithms, for it can achieve the best result in almost each scene only except fr3/sitting _half. Therefore, it can be summarized that our proposed method can effectively address the issue of static assumption failure in visual SLAM in dynamic scenes, thereby significantly improving its positioning accuracy and robustness.

**Table 3:** Comparison of pose estimation accuracy obtained by different SLAM systems in 8 different scenes.

Scene	Dyna-SLAM	DS-SLAM	MR-SLAM	DRSO-SLAM	OVD-SLAM	ISFM-SLAM
fr3/walking_static	**0.0060**	0.0081	0.0656	0.01112	0.0087	0.0081
fr3/walkig_xyz	**0.0150**	0.0247	0.0932	0.01576	0.1091	0.0164
fr3/walking_rpy	0.0350	0.4442	0.1333	0.07515	0.0317	**0.0301**
fr3/walking_half	0.0250	0.0303	0.1252	0.02684	0.3512	**0.0246**
fr3/sitting_static	None	**0.0064**	None	**0.0064**	0.0125	**0.0064**
fr3/sitting _xyz	0.0150	None	0.0482	None	0.0200	**0.0103**
fr3/sitting _rpy	None	None	None	None	0.0929	**0.0162**
fr3/sitting _half	0.0170	None	0.0470	None	**0.0147**	0.0175

In each scene, the best RMSE results of the ATE obtained by all compared SLAM systems are bolded.

### 4.4 Ablation studies

#### 4.4.1 Effectiveness of the modified components in the improved instance segmentation network

In the improved instance segmentation network proposed in this paper, we made two primary modifications to YOLACT: replacing the backbone with Res2Net-50 and using CIoU_Loss as the loss function. To thoroughly verify the effectiveness of these improvements, the ablation experiments in this subsection not only compare Res2Net-50 and CIoU_Loss with the original backbone and loss function used in YOLACT, but also with several other backbones and loss functions. The datasets used are still the COCO Minitrain dataset, and the experimental settings are also consistent with those described in Section 4.2. The results of the ablation experiments for the backbone and loss function are presented in Tables 4, 5, respectively.

**Table 4:** Ablation study of different backbones of instance segmentation network.

Backbone	FPS	mAP	AP50	AP75	APS	APM	APL
ResNeXt-50	34.2	28.20	49.61	30.15	11.32	33.94	42.89
ResNeSt-50	35.3	24.22	44.34	31.18	12.66	27.05	46.39
ResNet-101	42.0	29.91	48.62	31.32	10.06	31.44	47.85
Res2Net-50 (Ours)	42.2	33.61	56.24	36.26	16.47	36.21	49.82

**Table 5:** Ablation study of different loss functions of instance segmentation network.

Loss function	FPS	mAP	AP50	AP75	APS	APM	APL
Dice loss	33.10	28.90	48.01	31.52	9.46	30.87	47.14
EIOU loss	39.15	29.83	48.70	31.06	10.01	31.29	47.84
Smooth L1 loss	45.50	28.65	48.10	31.62	10.49	30.77	47.14
CIoU_Loss (Ours)	42.20	33.61	56.24	36.26	16.47	36.21	49.82

First, we replaced the backbone of the improved instance segmentation network with other ResNet-based architectures, including ResNet-101 (Bolya et al., 2019), ResNeXt-50 (Xie et al., 2017), and ResNeSt-50 (Zhang Y. H. et al., 2022; Zhang H. et al., 2022). Among these, ResNet-101 is the original backbone used by YOLACT. As shown in Table 4, the mAP of both ResNeXt-50 and ResNeSt-50 did not surpass that of Res2Net-50, or even ResNet-101. This is primarily because, although ResNeXt introduces greater parallel cardinality and ResNeSt employs split convolution strategies to enhance feature learning, they may not be as effective in multi-scale feature representation as Res2Net. This ultimately led to their poorer performance in instance segmentation tasks. For ResNet-101, its deeper architecture not only results in a slightly lower FPS compared to ResNet-50, but also leads to a reduction in mAP relative to Res2Net-50 primarily due to a certain degree of overfitting. Consequently, the experimental results presented in Table 4 demonstrate that employing Res2Net-50 as the backbone enables the improved instance segmentation network to achieve superior performance in terms of both segmentation accuracy and computational efficiency.

Second, we replaced the loss function of the improved instance segmentation network with Dice Loss (Li et al., 2019), EIOU Loss (Zhang Y. H. et al., 2022; Zhang H. et al., 2022), and Smooth L1 Loss (Bolya et al., 2019), where the last one is the loss function originally used in YOLACT. According to Table 5, the models employing Dice Loss and EIOU Loss show negligible improvements in segmentation accuracy compared to the model using Smooth L1 Loss. In contrast, the improved instance segmentation network using CIoU_Loss demonstrates a significant enhancement in AP-related metrics, albeit with a slight reduction in computational efficiency. The primary reason for the improvement is that CIoU_Loss considers not only the IoU overlap area but also the distance between center points and the aspect ratio. This enables CIoU_Loss to better handle challenging localization scenarios, resulting in substantially improved regression performance for the predicted bounding boxes compared to Smooth L1.

#### 4.4.2 Effectiveness of the PnP-based motion consistency detection method

**Figure 5:**
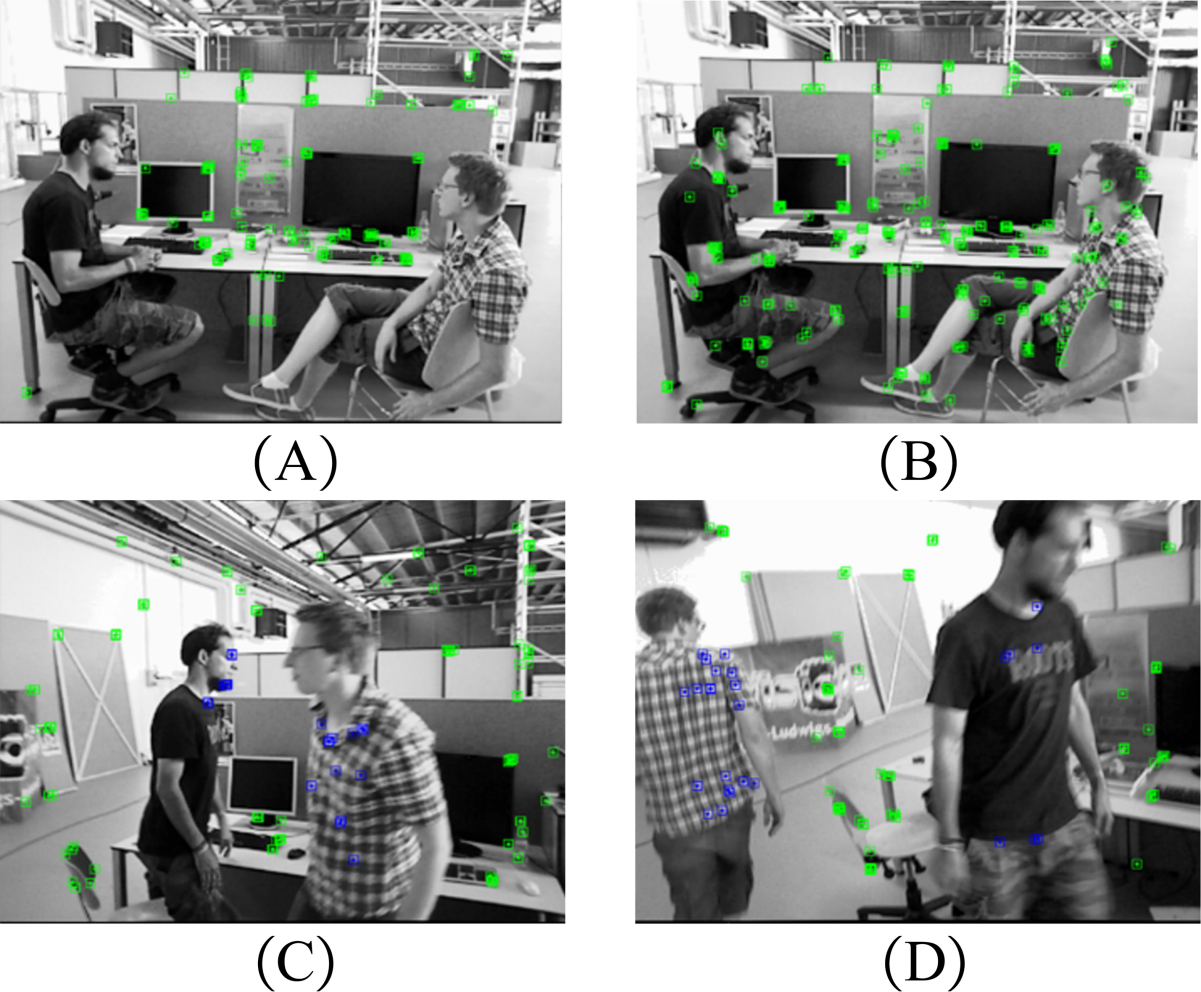
The feature point extraction result of the PnP-based motion consistency detection method combined with the improved instance segmentation network. **(A)** Result only by the improved YOLACT for a static sample. **(B)** Result by the motion consistency detection combined with improved YOLACT for a static sample. **(C,D)** Results by the motion consistency detection combined with improved YOLACT for samples containing people in motion. The raw images were obtained from the TUM Dataset, and this dataset is licensed under a Creative Commons 4.0 Attribution License (https://cvg.cit.tum.de/data/datasets/rgbd-dataset).

To verify the effectiveness of the proposed PnP-based motion consistency detection method, in this section, we employed this method combined with the proposed instance segmentation network on one static sample and two samples containing people in motion. The corresponding results of the feature point extraction of these samples are illustrated in Figures 5B–D, respectively, while Figure 5A is the feature point extraction result for a static sample when only the instance segmentation network is employed.

According to Figure 5A, it is evident that the feature points on the person in a static state are all removed and do not participate in the pose calculation. When the motion consistency detection algorithm and instance segmentation network are integrated and implemented, Figure 5B demonstrates that feature points on a stationary person can be successfully recovered. When the person in the sample is in a state of motion, as shown in Figures 5C,D, the feature points on the person can be removed (the blues points in Figures 5C,D). Therefore, it can be summarized that the motion consistency detection algorithm based on PnP can effectively remove the dynamic ORB feature points, and thus improve the accuracy of the camera pose.

#### 4.4.3 Effectiveness of the BEBLID feature matching

To verify the effect of the BEBLID descriptor on improving the feature matching accuracy of the proposed system, we compared the feature matching rate and computation time of the adopted BEBLID descriptor and the BRIEF descriptor in this section. These experiments were conducted on the adjacent image frames in two sets of low-dynamic sequences including fr3_stingting_static and fr3_stingting_rpy, as well as in two sets of high-dynamic sequences including fr3-walking_malf and fr3-walking_xyz, of the TUM dataset. A total of 500 feature points was extracted for each image frame, and then single response matrixes are employed to determine the number of matching points based on the Hamming distance. The RANSAC threshold was set to 3. The feature matching rate is defined as the percentage of the number of matching points.

**Figure 6:**
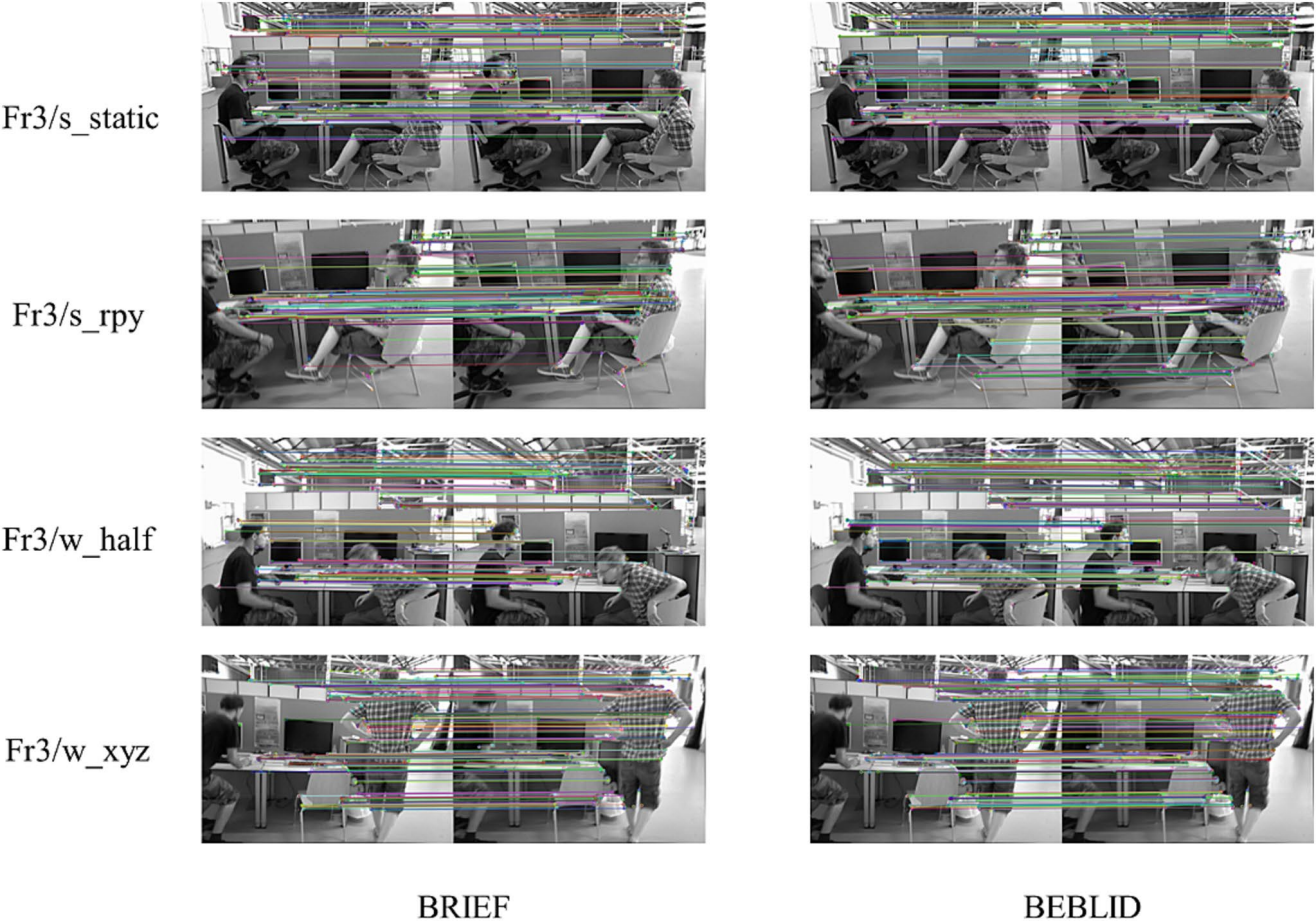
Comparison of specific matching results by the matching algorithms based on the BRIEF and BEBLID descriptors. The raw images were obtained from the TUM Dataset, and this dataset is licensed under a Creative Commons 4.0 Attribution License (https://cvg.cit.tum.de/data/datasets/rgbd-dataset).

Table 6 presents a comparison of the matching rates and the computational time by the matching algorithms based on the BEBLID and BRIEF descriptors across the selected four sequences. The average matching rates of the algorithm based on BEBLID descriptors are observed to be 6.6 and 6.9% higher than those of the algorithm based on BRIEF descriptors, respectively. Furthermore, BEBLID employs parallel computing to calculate each feature point descriptor, resulting in an average increase in calculation efficiency of 14.1 and 15.1%, respectively. Moreover, the specific feature matching results corresponding to each comparison in Table 6 are illustrated in Figure 6 to further demonstrate the effectiveness of the adopted descriptor. From Figure 6, it can be seen that on the same image frame, the matching algorithm based on the BEBLID descriptor has more correctly matched feature points than that based on the BRIEF descriptor, especially on some instances in the corners of the image.

**Table 6:** Comparison of matching rates and computational time by the matching algorithms based on the BRIEF and BEBILD descriptors on four sequences.

Dataset	descriptor	Matching number	Point number	Matching rate	Time /s
Fr3/sitting_static	BRIEF	389	348	89.4%	0.0373
	BEBLID	386	360	93.2%	0.0328
Fr3/sitting_rpy	BRIEF	345	254	73.6%	0.0341
	BEBLID	342	284	83.1%	0.0286
Fr3/walking_half	BRIEF	348	235	67.5%	0.0346
	BEBLID	337	267	79.2%	0.0291
Fr3/walking_xyz	BRIEF	338	256	75.7%	0.0332
	BEBLID	338	263	77.8%	0.0284

## 5 Real-world experiment and discussions

### 5.1 Experimental setup

**Figure 7:**
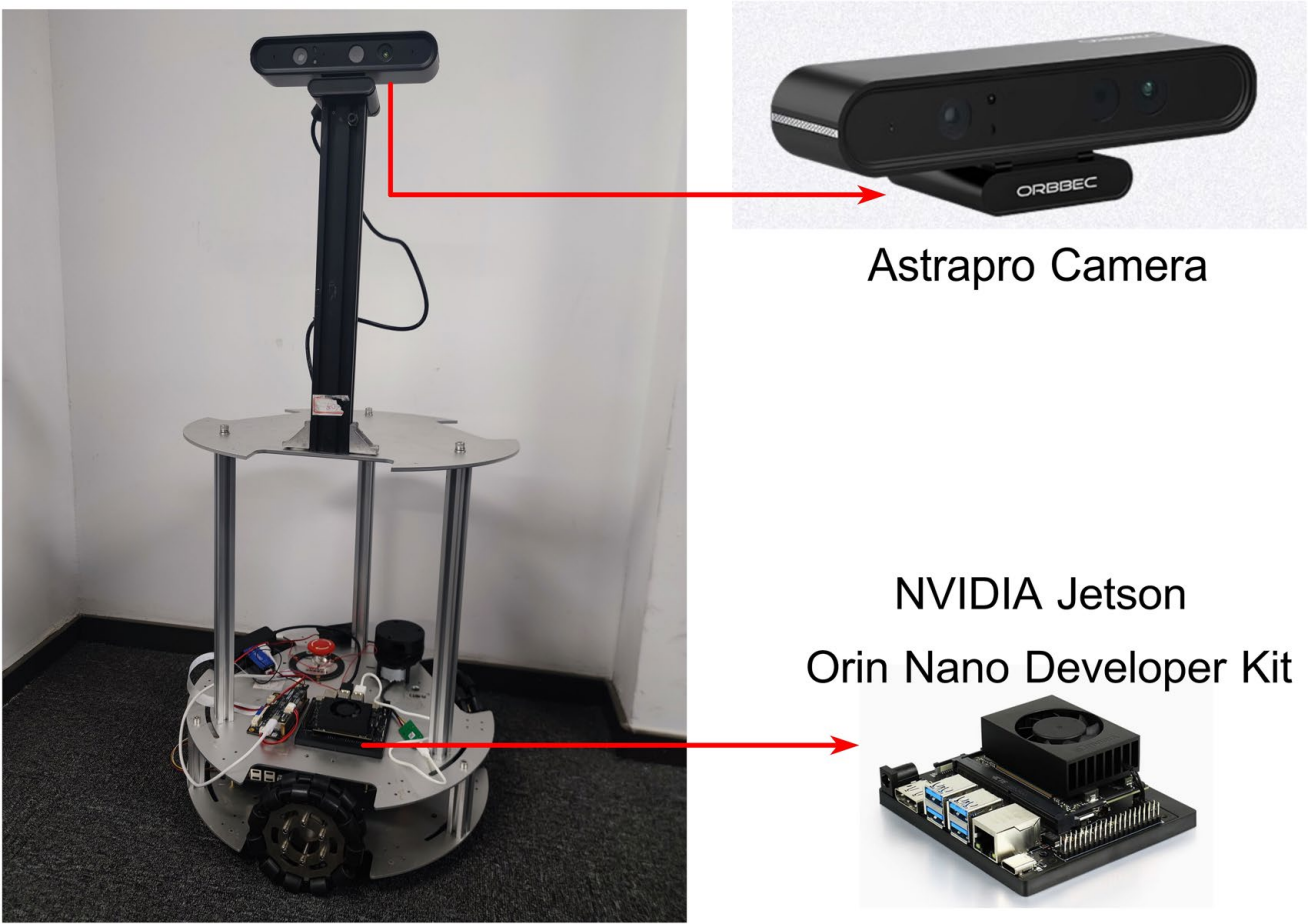
The three-wheeled mobile robot for the real-world experiment.

To evaluate the effectiveness of ISFM-SLAM in solving real-world tasks and its advantages over ORB-SLAM2, we deployed both the systems on a three-wheeled mobile robot for real-world experiments. As depicted in Figure 7, the mobile robot is equipped with an Astrapro RGB-D camera, capturing images at a frame rate of 30 FPS with a resolution of 640 × 480. ISFM-SLAM and ORB-SLAM2 were implemented on the NVIDIA Jetson Orin Nano Developer Kit of the robot, and both SLAM systems were initiated through the Robot Operating System (ROS). The parameters of the SGD optimizer for the instance segmentation network were adjusted for the experiment, with an initial momentum set to 0.9, a learning rate of 0.0001, and a weight decay coefficient of 0.00015.

### 5.2 Results and discussions

**Figure 8:**
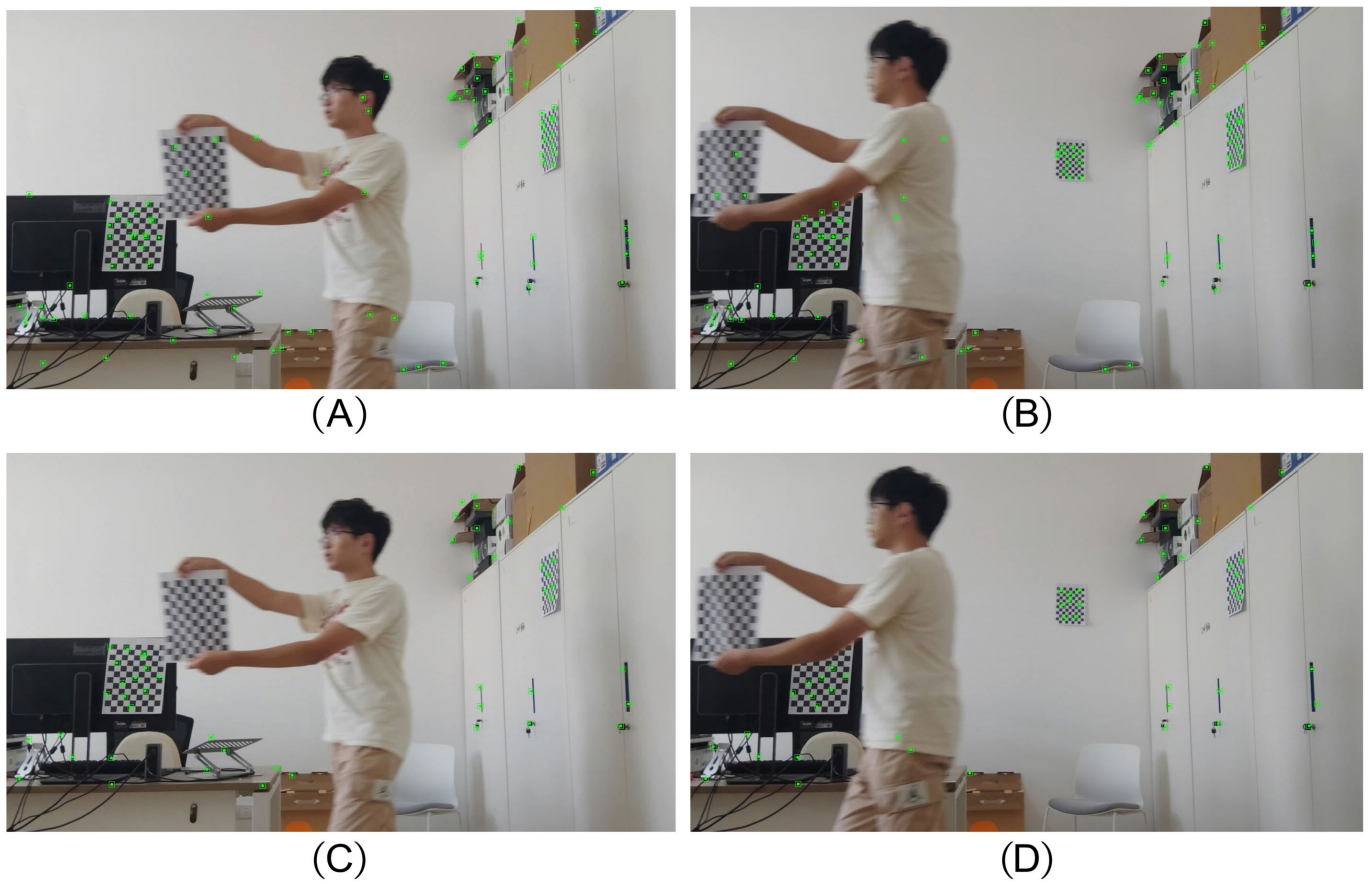
Experimental results in real environment. **(A,B)** Final preserved feature points by ORB-SLAM2; **(C,D)** Final preserved feature points by ISFM-SLAM.

To better emphasize the impact of our proposed improvements, we conducted an experiment where the robot remained stationary to capture moving people, testing the sensitivity of ISFM-SLAM and ORB-SLAM2 to dynamic objects, rather than merely scanning a static laboratory scene. As we did not have the necessary equipment to record ground-truth trajectories, the analysis focuses on how dynamic objects influence our SLAM system compared to its competitor. The experimental results are presented in Figure 8.

Figures 8A,B show that after running its tracking thread in a real laboratory scene, ORB-SLAM2 detected numerous feature points. However, ORB-SLAM2 fails to effectively exclude the influence of dynamic objects, such as the moving person in the images. In contrast, Figures 8C,D display the final retained feature points of ISFM-SLAM, clearly showing the absence of feature points on the moving person. This demonstrates that the PnP-based motion consistency detection can accurately distinguish between the motion and stationary states of objects, effectively removing feature points associated with moving objects. Simultaneously, our improved instance segmentation network accurately segments the moving person, preventing ISFM-SLAM from mistakenly removing feature points outside the segmentation boundary. Moreover, the retained feature points can be efficiently matched using the BEBLID feature matching approach, enabling ISFM-SLAM to achieve superior feature matching results.

## 6 Conclusion

This paper proposed a visual SLAM system named ISFM-SLAM for dynamic scenes based on the ORB-SLAM2 framework. To enhance the multi-sensory capabilities and prediction accuracy of the instance segmentation network, an improved YOLACT model was introduced into the ISFM-SLAM system, with the Res2Net model as its backbone and the CIoU_Loss as its loss function. Then, a PnP-based motion consistency detection approach is proposed to combined with the improved instance segmentation network, enabling the ISFM-SLAM system to effectively filter dynamic feature points. Moreover, the original BRIEF descriptor in the ORB-SLAM2 was replaced by the BEBLID descriptor to achieve efficient matching of ORB feature points. The simulation results demonstrate the effectiveness of the aforementioned improvements and the advantages of ISFM-SLAM over ORB-SLAM2 and other dynamic SLAM systems. Furthermore, real-world experiments conducted on mobile robots confirm that ISFM-SLAM can effectively mitigate the impact of dynamic objects during mapping, proving its feasibility in practical applications. In the future, we will lightweight the instance segmentation network proposed in this paper to improve its real-time performance, and modify the BEBLID descriptor so that the SLAM system can be implemented in more complex dynamic scenes.

## Data Availability

Publicly available datasets were analyzed in this study. This data can be found here: The TUM Dataset (Download Link): https://cvg.cit.tum.de/rgbd/dataset/. The COCO Minitrain dataset (Download Link): http://images.cocodataset.org/zips/train2017.zip and http://images.cocodataset.org/zips/val2017.zip.
